# Comparative Effectiveness of Intravitreal Anti-Vascular Endothelial Growth Factor Therapies for Managing Neovascular Age-Related Macular Degeneration: A Meta-Analysis

**DOI:** 10.3390/jcm11071834

**Published:** 2022-03-25

**Authors:** Frédéric Matonti, Jean-François Korobelnik, Corinne Dot, Vincent Gualino, Vincent Soler, Sarah Mrejen, Marie-Noëlle Delyfer, Stéphanie Baillif, Maté Streho, Pierre Gascon, Catherine Creuzot-Garcher, Laurent Kodjikian

**Affiliations:** 1 Centre Monticelli Paradis, 433 Bis Rue Paradis, 13008 Marseille, France; pierre.gascon3@gmail.com; 2 National Center for Scientific Research (CNRS), Timone Neuroscience Institue (INT), Aix Marseille University, 13008 Marseille, France; 3 Groupe Almaviva Santé, Clinique Juge, 13008 Marseille, France; 4 Department of Ophthalmology, Bordeaux University Hospital, 33000 Bordeaux, France; jean-francois.korobelnik@chu-bordeaux.fr (J.-F.K.); marie-noelle.delyfer@chu-bordeaux.fr (M.-N.D.); 5 INSERM, BPH, UMR1219, Bordeaux University, 33000 Bordeaux, France; 6 Department of Ophthalmology, Desgenettes Military Hospital, 69003 Lyon, France; corinnedot.pro@hotmail.fr; 7 Clinique Honoré Cave, Department of Ophthalmology, 82000 Montauban, France; vincent.gualino@gmail.com; 8 Unité de Rétine, Ophthalmology Department, Hôpital Pierre-Paul Riquet, Toulouse University Hospital, 31300 Toulouse, France; vincesoler@yahoo.fr; 9 Place Baylac, TSA 40031, CEDEX 9, 31059 Toulouse, France; 10 Ophthalmology Department, AP-HP, Hôpital Lariboisière, Université de Paris, 75014 Paris, France; 11 University Toulouse III, 31000 Toulouse, France; 12 CERCO UMR 5549, Centre National de la Recherche Scientifique, 31000 Toulouse, France; 13 Centre d’Imagerie et de Laser, 75015 Paris, France; sarahmrejen.uretsky@gmail.com; 14 Centre Hospitalier National Ophtalmologique des 1520, 75012 Paris, France; 15 Department of Ophthalmology, Pasteur 2 University Hospital, Côte d’Azur University, 06108 Nice, France; baillif.s@chu-nice.fr; 16 Explore Vision Centre, 75001 Paris, France; mstreho@yahoo.fr; 17 Department of Ophthalmology, Lariboisière Hospital, 75010 Paris, France; 18 Department of Ophthalmology, Aix-Marseille University, Hôpital Nord, Chemin des Bourrely, 13008 Marseille, France; 19 Department of Ophthalmology, University Hospital, CHU Dijon, 21000 Dijon, France; catherine.creuzot-garcher@chu-dijon.fr; 20 Department of Ophthalmology, Croix-Rousse University Hospital, Hospices Civils de Lyon, 69002 Lyon, France; laurent.kodjikian@chu-lyon.fr; 21 UMR-CNRS 5510 Matéis, University of Lyon, 69622 Villeurbanne, France

**Keywords:** age-related macular degeneration (AMD), aflibercept, comparative therapies, effectiveness, intravitreal anti-vascular endothelial growth factor, meta-analysis, ranibizumab, treat-and-extend, pro re nata regimen

## Abstract

Intravitreal injections (IVI) of anti-vascular endothelial growth factor (anti-VEGF) have become the standard of care for age-related macular degeneration (AMD). Although most pivotal trials have used monthly injections, alternative strategies that enable the injections to be administered on a more flexible schedule, including pro re nata (PRN) and treat-and-extend (T&E) regimens, are being applied more frequently. This review sought to provide further scientific evidence about the visual outcomes and treatment burden among the currently available anti-VEGF agents and regimens, including aflibercept, ranibizumab, abicipar and brolucizumab. To this end, a systematic review of published randomized studies was conducted from the MEDLINE and EMBASE databases and the Cochrane library, and a meta-analysis was applied to the obtained data using single-means modeling to compare the efficacy and maintenance among the different available treatments and regimens at Years 1 and 2. Quality analysis identified the best-informed data for modeling purposes. Overall, 47 relevant publications were retrieved for the analysis. Superior efficacy, meaning that there were observed improvements in visual acuity (VA) and central retinal thickness (CRT), occurred with monthly versus PRN regimens, yet a higher IVI number was also observed. Conversely, the T&E regimens displayed similar efficacy to the monthly regimens, but with a reduced IVI number. Aflibercept T&E exhibited similar efficacy to ranibizumab T&E, but with significantly lower IVI numbers at both Year 1 (*p* < 0.0001) and Year 2 (*p* = 0.0011). Though all of the regimens resulted in maintained efficacy between Years 1 and 2, the required IVI number varied. The retrieved data did not enable other regimens or newer anti-VEGF agents such as brolucizumab to be compared. In conclusion, the T&E regimens were shown to be the most efficient, optimizing durable effectiveness whilst minimizing the IVI number in newly diagnosed exudative AMD, with aflibercept requiring the lowest IVI number.

## 1. Introduction

Neovascular age-related macular degeneration (nAMD) is a progressive degenerative disease of the retinal macula that can lead to permanent central vision impairment and blindness [1]. The overall prevalence of the condition in developed countries is estimated to be approximately 8.7% [2]. Risk factors for developing AMD include smoking, a higher body mass index and hypertension, as well as soft drusen and pigment abnormalities within the macula [3,4]. Over the past decade, substantial improvements have been made in the treatment of nAMD, following the introduction of new molecules and treatment regimens. These comprise several agents that are targeted at vascular endothelial growth factor (VEGF), including ranibizumab, bevacizumab, aflibercept, abicipar pegol and brolucizumab.

Indeed, VEGF upregulation, which is assumed to be induced by retinal and choroidal hypoxia, has been shown to drive angiogenesis and increase vascular permeability, which has been observed in nAMD [2]. The visual acuity (VA) gain reported in clinical trials using anti-VEGF agents during the first two years of treatment [5] resulted in a marked upgrade in disease prognosis [6], thereby establishing anti-VEGF agents as the standard of care for this condition [7]. Despite considerable improvements in legal blindness related to nAMD following anti-VEGF introduction, real-world studies have failed to confirm these encouraging data, which is primarily due to under-treatment and a lack of long-term results [2]. A study using the Fight Retinal Blindness observational registry showed that it was possible to achieve good visual outcomes in eyes managed in routine clinical practice with a treat-and-extend (T&E) regimen while also decreasing the treatment burden and number of clinic visits at the same time [8]. Additionally, the monthly intravitreal anti-VEGF injections that are required for the standard of care have been proven to be burdensome for both patients and healthcare systems, often leading to poor treatment adherence in real-life practice [9]. Thus, other regimens that administer anti-VEGF agents at various fixed intervals, such as every 8 or 12 weeks (q8w and q12w, respectively) or as-needed (pro re nata [PRN]), as well as T&E regimens with treatment intervals based on individual disease activity, have been investigated [1,10].

To compare different anti-VEGF agents and regimens, several meta-analyses have been performed [11,12,13,14] with the goal of better assisting physicians in their therapeutic decision-making process. Indeed, meta-analyses primarily seek to provide more accurate outcomes and comprehensive conclusions than individual studies through the use of a pool of previously published studies that allow for data comparison [15]. It was in this context that a Cochrane library meta-analysis demonstrated the similar effectiveness and safety of aflibercept versus ranibizumab for managing nAMD patients [12]. Another Cochrane library meta-analysis by Solomon et al. indicated that anti-VEGF agents such as pegaptanib, ranibizumab and bevacizumab had similar effectiveness [11]. A recent network meta-analysis indirectly compared aflibercept and ranibizumab administered via T&E regimens and revealed a similar efficacy between the treatment arms, but for aflibercept, this was associated with a significantly reduced number of injections over a two-year period [14]. Nevertheless, it must be stressed that no meta-analysis conducted on nAMD has compared all of the anti-VEGF agents and treatment regimens that are currently available. It must similarly be stressed that 10 to 35% of patients either failed to respond to anti-VEGF agents, referred to as non-responders, or developed resistance to anti-VEGF agents over time, a condition known as tachyphylaxis [16,17]. Given this background, our review primarily sought to provide further scientific evidence regarding the visual outcomes and treatment burden among all of the available anti-VEGF agents and regimens. To this end, we performed a meta-analysis that included data from all of the randomized clinical trials conducted in the nAMD field that were designed to (1) compare the effectiveness of the available treatment regimens at Years 1 and 2, (2) assess the efficacy maintenance of the various regimens over time and (3) compare the effectiveness of various anti-VEGF agents using the same treatment regimen at Years 1 and 2.

## 2. Methods

### 2.1. Selection of Studies

A systematic literature review was performed according to the Preferred Reporting Items for Systematic Reviews and Meta-Analyses (PRISMA) guidelines and the Cochrane Collaboration statement [18,19]. We looked for studies using a sensitive search strategy pertaining to anti-VEGF agents used in AMD management in the PubMed/Medline^®^, Embase^®^ (access via Ovid platform) and Cochrane CENTRAL databases from the earliest available time up to 16 March 2020. Moreover, studies from ClinicalTrial.gov (accessed on 15 March 2022). with detailed results that had not been published elsewhere were retrieved, as well. The individual search terms applied for the searches are listed in Table 1. The selection of search terms was based on a two-expert consultation. Studies from PubMed were limited to “clinical trials” and “human” studies, with animal and basic science studies being excluded from the analysis, whereas studies from ClinicalTrials.gov were limited to those that were either “terminated” or “completed”. After the database search, two of the authors of this review independently selected articles, and selection was followed by a double-check and validation process. The manual selection process for eligible publications comprised a review of the titles and abstracts; studies that did not match the previously established criteria were excluded. Next, the abstracts and full texts of the retrieved publications were read by the authors in full, thereby confirming their eligibility. The selected publications reported results of prospective, randomized controlled studies related to exudative nAMD and its synonyms and acronyms. The Cochrane risk of bias tool was applied to further assess the quality of the included studies. All of the selected studies had at least one treatment arm involving first-line treatment, thus involving treatment-naïve patients undergoing first-line treatment with one of the following agents: aflibercept 2 mg, ranibizumab 0.5 mg, bevacizumab 1.25 mg, brolucizumab 6mg or abicipar pegol 2 mg. Phase 1 studies, in addition to subgroup or post hoc analyses, were excluded. Moreover, to meet the eligibility criteria, these studies needed to include primary or secondary outcome measures assessing the best-corrected visual acuity (BCVA) and BCVA at ≥12 months after the baseline. In contrast, studies involving previously treated patients and those with a follow-up duration of <12 months or >24 months without any available intermediate data were not considered. Based on these criteria, a total of 69 studies were retrieved.

### 2.2. Data Extraction and Outcome Analyses

Relevant data were manually extracted from each selected publication and were transcribed into an Excel sheet, and these data were subsequently verified by a second reader. The following data were included: population-related criteria, such as the number of patients, patients lost to follow-up and demographic data; choroidal neovascularization (CNV) features such as the type and presence of polypoidal choroidal vasculopathy (PCV); treatment criteria, including the therapeutic regimen, proportion of patients receiving treatment every 4, 6, 8, 10, 12 or 16 weeks, proportion of patients receiving treatment every ≥8, 12 or 16 weeks, average treatment interval at month 12 or 24 and the IVI number; functional criteria, including the BCVA at the baseline and at month 12 or 24, mean gain in BCVA from the baseline or from randomization depending on the trial design, proportion of patients with BCVA gain ≥15 letters, proportion of patients with BCVA gain ≥5 letters and proportion of patients with VA loss ≥15 letters; anatomical features such as central retinal thickness (CRT) at the baseline and at month 12 or 24, mean reduction in CRT and the intra-retinal fluid (IRF) or subretinal fluid (SRF) at the baseline and at month 12 or 24.

To define the follow-up duration, the outcomes that were assessed at 52 ± 4 weeks were considered one-year data, and those assessed at 104 weeks ± 4 weeks were considered two-year data; study arms with other follow-up durations were excluded from the meta-analysis. For better homogeneity within each protocol type and to ensure relevant comparisons, only study arms with an induction phase consisting of the administration of at least two monthly loading doses were considered. In the event of a change in the treatment/regimen during the second year, two-year data were excluded from the analysis. For every six-weekly (q6w) dosing regimen, study arms that were in the induction phase or that took place throughout the treatment period were excluded, meaning loading dose and maintenance treatment regimens could be distinguished during the second year. When the studies were selected, it is important to note that bevacizumab was being used off-label for the treatment of wAMD in many European countries, and the question of whether the use of this off-label therapy was responsible or not went unanswered, with a benefit–risk assessment under investigation [20]. Therefore, study arms involving the off-label use of bevacizumab alone were not retained for the final analysis. Of note, the European Medicines Agency (EMEA) recently documented that it did not approve a marketing authorization for a bevacizumab-based drug due to the lack of appropriate safety and efficacy that had been demonstrated and concerns about the risks outweighing the benefits. Instead, a temporary-use authorization (TUA) was granted [21].

### 2.3. Data Synthesis and Analysis

Studies were assessed for clinical and methodological heterogeneity and for the risk of bias, as well as to determine their suitability for inclusion in the model. Single-means modeling based on fixed- and random-effect models and heterogeneity tests were applied to compare the anti-VEGF regimens, as well as agents within the same regimen. Next, “2 to 2” modeling was applied to further refine the comparisons of various anti-VEGF regimens and agents, aiming to retain the most relevant and robust data. A meta-analysis of the data was conducted to compare the study arms. All of the study arms with means, standard deviations and counts were entered into the model to attain significant statistical power. Intra-study comparisons between randomized study arms were not carried out. Visual outcome data were analyzed following conversion into ETDRS letters if not already available, similar to in [22].

Data quality analysis was conducted to identify the best-informed data at Years 1 and 2, as well as the best-informed clinical outcomes at Year 1, for inclusion in the model. For illustration, the age criterion was very well-informed, whereas other criteria such as the number of visits were poorly informed, meaning that the latter were unable to provide statistical power. The best-informed data at Years 1 and 2 were comparisons of monthly, T&E and PRN regimens, as well as comparisons of aflibercept and ranibizumab, with 75% of the eye of concern being included. Moreover, the best-informed clinical outcomes at Year 1 were improvements in BCVA, reductions in CRT and the IVI number. All of the statistical analyses were conducted using R statistical software version 3.6.0 (R Core Team 224 2019).

### 2.4. Compliance with Ethical Guidelines

This meta-analysis was a secondary statistical analysis that combined data collected by eligible clinical trials. The data sources primarily included data from the public domain, and one clinical trial contained limited patient data. All of the clinical data were anonymized before they were entered into the model, with the analysts having no access to any personal information that would enable them to identify individual patients. For this reason, ethical committee approval was not required for this meta-analysis.

## 3. Results

### 3.1. Included Studies

**Figure 1:**
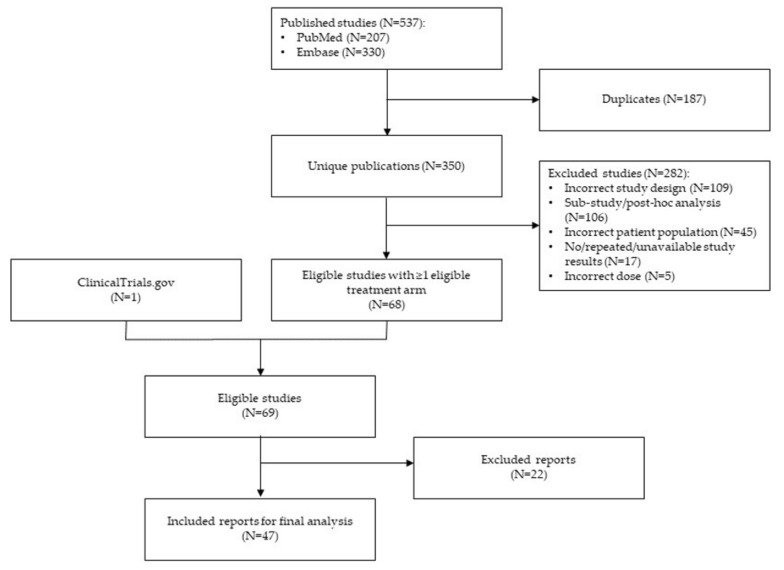
Flow chart of included reports.

The literature searches across all of the databases and the references of systemic reviews returned a total of 5389 references. After further filtering, a total of 207 publications were retrieved from PubMed, and 330 were retrieved from Embase. The removal of 187 duplicates yielded 350 unique results. After further evaluation, 68 publications and one additional completed clinical trial with relevant results identified from ClinicalTrials.gov were selected. Following the application of previously mentioned inclusion and exclusion criteria to these 69 reports, 47 were retained for the final analysis (Figure 1), and the details of these 47 reports are provided in Table 2. Overall, the included publications provided one-year data from 78 treatment arms (12,689 eyes) and two-year data from 35 treatment arms (8560 eyes).

### 3.2. Evaluated Treatments

Among the studies included in the analysis, the following anti-VEGF agents were evaluated: ranibizumab 0.5 mg (74% of studies), aflibercept 2 mg (20% of studies) and brolucizumab 6 mg (4% of studies), whereas none of the included studies evaluated abicipar pegol 2 mg. Three studies using bevacizumab 1.25 mg alone were not retained. For seven other studies using bevacizumab along with a comparative arm, the latter arm was considered for analysis, whereas the bevacizumab arm was not.

### 3.3. Comparison of Monthly versus Pro Re Nata (PRN) Regimens

**Figure 2:**
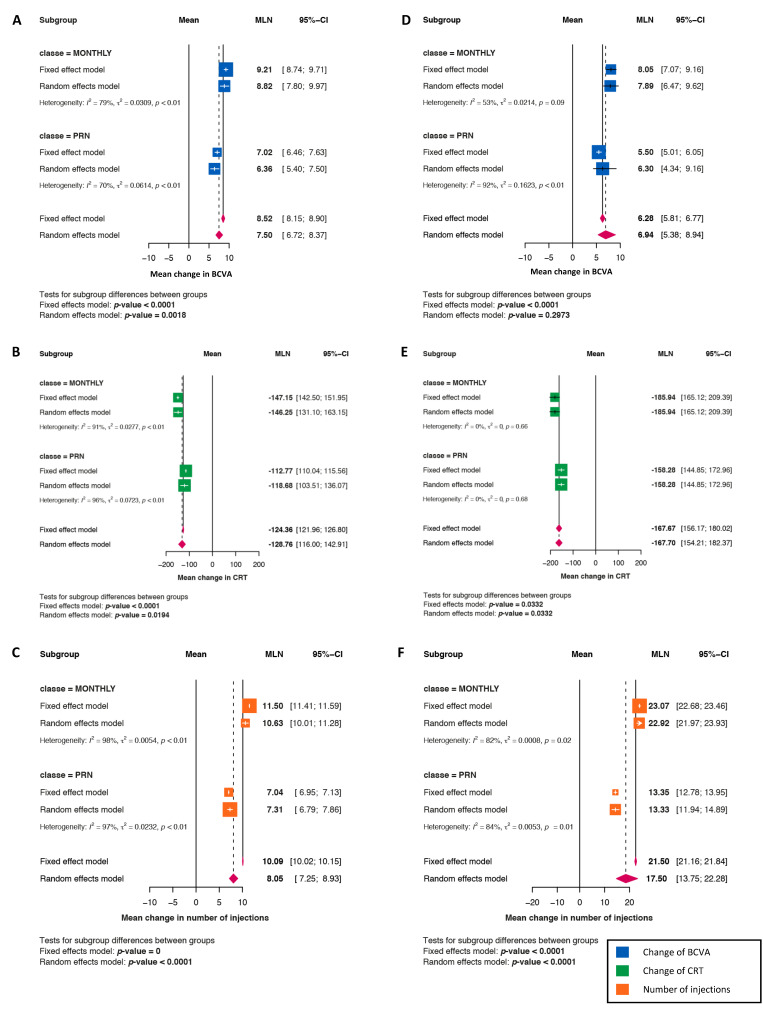
As needed (PRN) versus Monthly regimens. (**A**–**C**): Mean change at 1 year, in best corrected visual acuity ((**A**), blue squares), in CRT ((**B**), green squares) and in the number of injections ((**C**), orange squares). (**D**–**F**): Mean change at 2 years, in best corrected visual acuity ((**D**), blue squares), in CRT ((**E**), green squares) and in the number of injections ((**F**), orange squares).

For the comparison of monthly versus PRN regimens, at Year 1, the mean gains in the BCVA (8.82 vs. 6.36 letters; *p* = 0.0018) and the reductions in the CRT (mean −146.25 vs. −118.68 μm; *p* = 0.0194) were significantly higher with the monthly regimens than they were with the PRN regimens (Figure 2A, Figure 2B, respectively), and the IVI number was significantly higher with the monthly regimens (mean 10.6 vs. 7.3; *p* < 0.0001) (Figure 2C). At Year 2, the IVI number was significantly higher for the monthly regimens versus the PRN regimens (22.9 vs. 13.3; *p* < 0.0001), and the monthly regimens were associated with significantly greater reductions in the CRT (mean −185.94 vs. −158.28 μm; *p* = 0.0332) (Figure 2E,F). Concerning gains in the BCVA (mean 7.89 vs. 6.30 letters; *p* = 0.2973), no significant differences were identified between the monthly and PRN regimens (Figure 2D).

### 3.4. Comparison of Monthly versus Treat-and-Extend (T&E) Regimens

**Figure 3:**
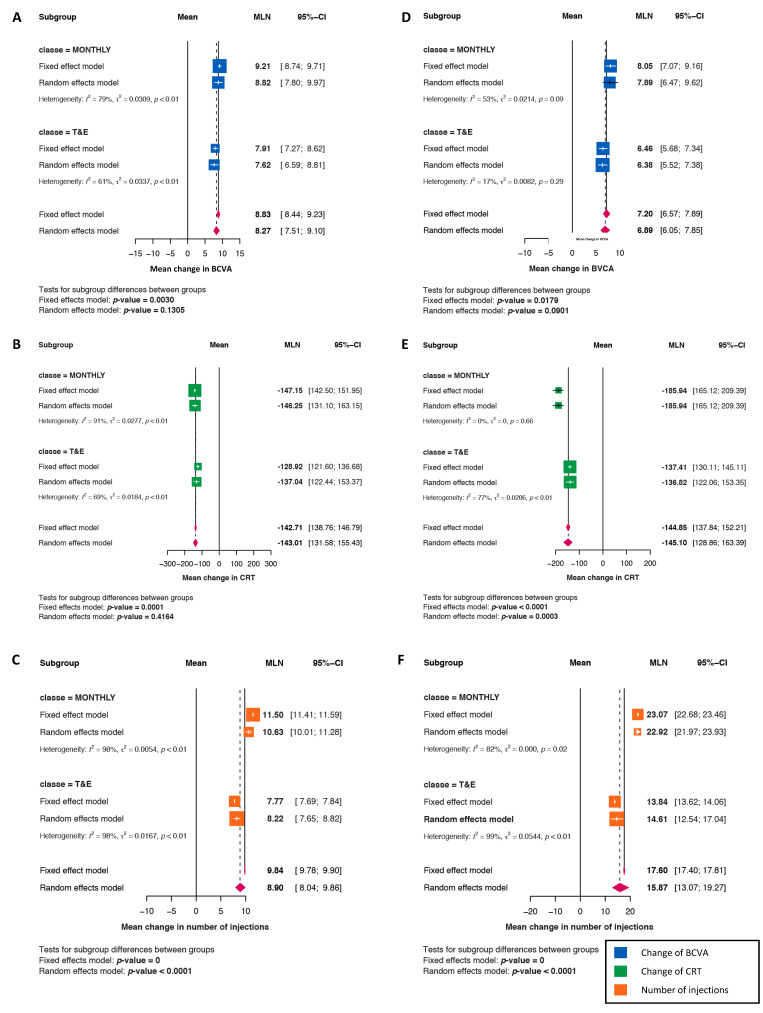
Treat and Extend (T&E) versus Monthly regimens. (**A**–**C**): Mean change at 1 year, in best corrected visual acuity ((**A**), blue squares), in CRT ((**B**), green squares) and in the number of injections ((**C**), orange squares). (**D**–**F**): Mean change at 2 years, in best corrected visual acuity ((**D**), blue squares), in CRT ((**E**), green squares) and in the number of injections ((**F**), orange squares).

At Year 1, the improvements that were observed in the BCVA (8.82 vs. 7.62 letters; *p* = 0.1305) and CRT (mean −146.25 vs. −137.04 μm; *p* = 0.4164) were proven to be similar to the improvements observed in the monthly and T&E regimens, whereas the IVI number was significantly lower in the T&E regimens (10.6 vs. 8.2; *p* < 0.0001) (Figure 3). At Year 2, no differences in BCVA gains (mean 7.89 vs. 6.38 letters; *p* = 0.0901) were revealed between monthly and T&E regimens, but the reduction that was observed in CRT was significantly higher with the monthly than the T&E regimens (−185.94 vs. −136.82 μm; *p* = 0.0003), and the IVI number was still significantly lower with the T&E regimen (mean 22.9 vs. 14.6; *p* < 0.0001) (Figure 3).

### 3.5. Comparison of Pro Re Nata (PRN) Versus Treat-and-Extend (T&E) Regimens

At both Years 1 and 2, the improvements that were observed in the BCVA and CRT, as well as in the IVI number, were similar between the PRN and T&E regimens. The mean gains in the BCVA with PRN regimens versus with T&E regimens at Years 1 and 2 were 6.36 vs. 7.62 letters (*p* = 0.1075) and 6.30 vs. 6.38 letters (*p* = 0.9504), respectively. The corresponding mean reductions in CRT were −118.68 vs. −137.04 μm (*p* = 0.1116) and −158.28 vs. −136.82 μm (*p* = 0.048), whereas the IVI numbers were 7.3 vs. 8.2 (*p* = 0.0241) and 13.3 vs. 14.6 (*p* = 0.3409) at Years 1 and 2, respectively.

### 3.6. Maintaining Improvements over Time

The monthly, PRN and T&E regimens were all likely to maintain or even improve letter-gaining for BCVA and drying up for CRT between Years 1 and 2 (Table 3). Nevertheless, the mean IVI number differed with each of the regimens over time (10.6 and 22.9 for monthly regimens, 7.3 and 13.3 IVI for PRN regimens and 8.2 and 14.6 for T&E regimens at Years 1 and 2, respectively).

### 3.7. Comparing Aflibercept and Ranibizumab Treat-and-Extend (T&E) Regimens

**Figure 4:**
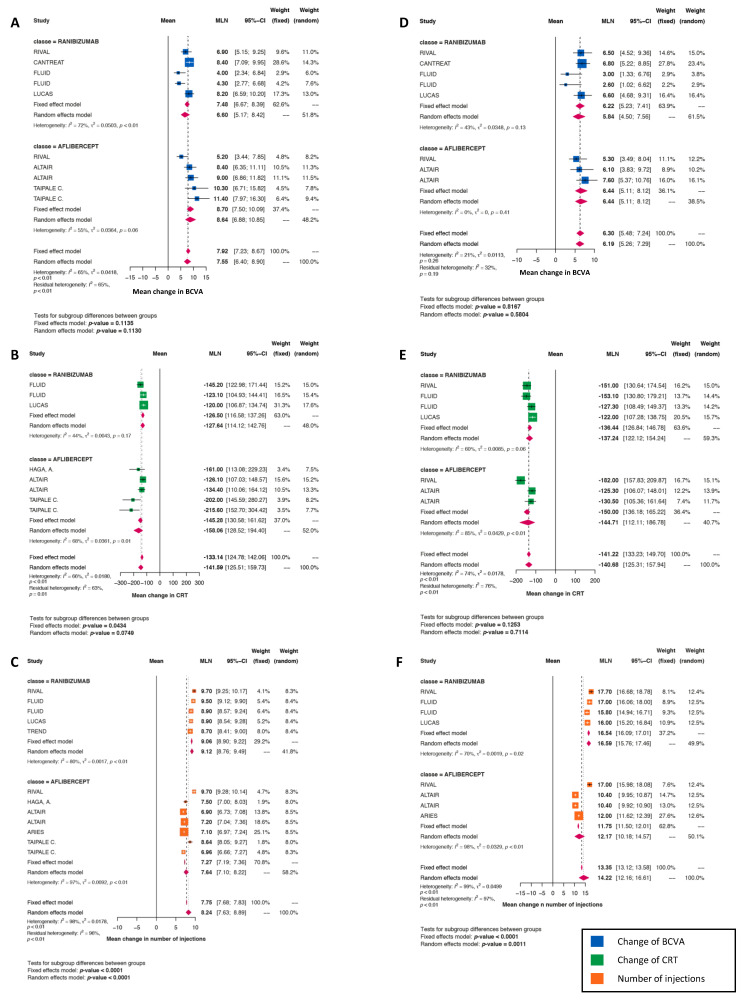
T&E regimen, Aflibercept versus Ranibizumab. (**A**–**C**): Mean change at 1 year, in best corrected visual acuity ((**A**), blue squares), in CRT ((**B**), green squares) and in the number of injections ((**C**), orange squares). (**D**–**F**): Mean change at 2 years, in best corrected visual acuity ((**D**), blue squares), in CRT ((**E**), green squares) and in the number of injections ((**F**), orange squares).

The mean gains in the BCVA (Figure 4) and reductions in CRT (Figure 4) were similar between aflibercept and ranibizumab T&E regimens at Years 1 and 2, whereas the mean IVI number was significantly lower with aflibercept than ranibizumab at both Years 1 (7.6 and 9.1, respectively) (*p* < 0.0001) and 2 (12.2 and 16.6, respectively) (*p* = 0.0011; Figure 4).

## 4. Discussion

Anti-VEGF agents are not a curative therapy, but they have become a mainstay and pillar in nAMD management. Indeed, these agents have been proven to slow down disease progression and partially reverse visual impairment [11,66]. Nevertheless, the need for repeated IVI has been a serious burden in AMD management for patients and healthcare systems alike. Hence, alternative administration regimes have been sought, with PRN and T&E regimens the most promising. Under a PRN regimen, the decision to provide an anti-VEGF injection is made at each visit based on the outcomes of the optical coherence tomography measurements. In contrast, when a T&E regimen is applied, the patient undergoes an IVI at each visit, with the between-visit intervals adjusted depending on the disease progression [14]. The primary objective of our meta-analysis was to retrieve further scientific data concerning the visual outcomes and treatment burden with different anti-VEGF agents provided according to either the standard monthly regimen or one of two alternative strategies, PRN or T&E, taking a two-year perspective.

In line with previously published data from the majority of relevant studies, positive outcomes were recorded for all of the anti-VEGF agents and regimens. To summarize our outcome data, at Year 2, both the monthly and T&E regimens resulted in CRT improvements, while the VA outcome improvements were maintained over time with all of the regimens (monthly, PRN, and T&E), and similar improvements in the VA and CRT outcomes were attained with the aflibercept regimens versus the ranibizumab T&E regimens. Hence, our meta-analysis has further confirmed certain previously published observations. Indeed, t significantly improved VA and CRT at Years 1 and 2 were previously reported with monthly versus PRN regimens, similar improvements in VA and CRT were reported at Year 1 with both monthly and T&E regimens and similar improvements were observed in the VA and CRT with PRN and T&E regimens, a finding that was revealed in prior meta-analyses [11,12,66]. In addition to previously published data, our analyses revealed that significantly fewer injections were required for PRN and T&E regimens versus monthly regimens at both time points. All in all, these results clearly suggest that T&E is the best alternative strategy that can be recommended for AMD management compared to the monthly and PRN strategies. Considering this T&E regimen, aflibercept generated similar results compared to ranibizumab, but it required a lower IVI number, meaning that this regimen is also able to provide a longer response. In this context, it must be stressed that a recent survey of retinal specialists found that two-thirds of the respondents in the United States (n = 586) and one-third of the respondents in Europe (n = 424) indicated a clear preference for the T&E regimen with respect to managing their treatment-naïve AMD patients (American Society of Retina Specialists’ “2015 global trends in retina” survey results).

The superior efficacy of VA and CRT obtained with monthly versus PRN regimens, as well as the similar efficacy observed with monthly versus T&E regimens in our analysis, are perfectly in line with a recently published Cochrane meta-analysis [67]. These authors revealed that newly diagnosed AMD patients receiving monthly anti-VEGF injections exhibited a slightly better VA at Year 1 versus those receiving PRN injections, whereas no difference in VA was found compared to the T&E regimen. Here, it must be mentioned that, according to the authors, the patients receiving monthly injections actually had a higher IVI number than those receiving injections via PRN or T&E regimens, meaning that patients who were treated monthly were exposed to an increased, although rare, risk of severe undesirable effects such as infections.

An interesting novelty of the current meta-analysis has been its ability to compare the long-term efficacy data of the various anti-VEGF and regimens. For this same purpose, other authors have already focused their research on the outcome data recorded at Years 1 and 2 [11,13,14,50,66]. According to their research, the PRN and T&E regimens displayed VA with decent stability and good control, as well as CRT improvements for up to two years, a finding that is perfectly in line with the monthly standard of care. Moreover, at Year 2, fewer injections were required with both regimens. Here, it must nevertheless be stressed that the PRN regimen appears to be more difficult to handle in real-life settings versus the T&E regimen due to the required monthly visits. In this context, it must be stressed that PRN regimens have been proven to be effective in prospective randomized studies with carefully selected patients and well-controlled conditions, whereas these regimens were reported to be poorly reproducible in real-life practice [9].

When comparing aflibercept and ranibizumab T&E regimens, their efficacy turned out to be quite similar, whereas this good outcome was achieved with a significantly lower IVI when aflibercept was used at both Years 1 and 2 (7.6 versus 9.1 at Year 1, and 12.2 versus 16.6 at Year 2). In practical terms, this means that aflibercept prolonged the IVI intervals. This outcome is in accordance with the results from a meta-analysis conducted by Ohji et al., which revealed that, at Year 2, aflibercept T&E was associated with six fewer injections on average compared to ranibizumab T&E, providing comparable visual improvements [14]. For clinical practice, this reduced IVI number likely represents a real advantage of aflibercept T&E, especially since anti-VEGF therapy is known to impose a significant additional constraint on patients due treatment-related anxiety and practical problems such as transport burden and common clinic visits [14].

With respect to our analytical methodology, single-means analysis is a new and interesting approach that deserves to be mentioned. This technique enabled us to conduct such a meta-analysis involving large amounts of data to compare three regimens (monthly, PRN and T&E) and two anti-VEGF agents (aflibercept and ranibizumab). Despite the strengths of this statistical methodology, the major limitation was the choice to exclude bevacizumab from the equation. Off-label use of bevacizumab has been discontinued from real-life use. Therefore, our analyses were limited by insufficient data availability. For this reason, several scenarios could not be properly analyzed. Hence, in our literature search, we only came across a few anti-VEGF agent comparisons between monthly versus PRN regimens because most comparisons concerned bevacizumab. It would be interesting to repeat this analysis while including bevacizumab. Likewise, we only identified very few comparisons within the q8w/q12w regimens, given that only brolucizumab has been administered according to the q8w regimen and only very limited data pertaining to the q12w regimen are available. With respect to the q8w regimen, scarcely any data were recorded during Year 2, with several study designs including a regimen change between Years 1 and 2.

We must emphasize several limitations to our report. A major drawback of our meta-analysis, which is rather common, is the failure to obtain data on all patients and from all trials. This may result in an acquisition bias, given that missing studies or patients may not be missing completely at random, resulting in biased outcomes. While meta-analyses represent a powerful tool for the design of future research and to provide evidence for the regulatory process, they may also be controversial, as several conditions are critical to a sound meta-analysis, and small violations of such conditions may lead to misleading results. Another drawback that should be mentioned is that there was a slight discrepancy between the time points at which the Year 2 data were collected.

## 5. Conclusions

This new meta-analysis revealed superior efficacy, reflected by improvements in VA and CRT with both monthly PRN regimens, but this improved efficacy was achieved with a higher IVI number. On the other hand, the T&E regimens demonstrated similar efficacy to the monthly regimens, but with a reduced IVI number. When comparing the T&E regimens of aflibercept and ranibizumab, aflibercept was associated with a reduced IVI number compared with ranibizumab, but the recorded parameters showed similar efficacy.

## Figures and Tables

**Table 1:** Search terms used in the systematic literature review.

Queried Database	Filter	No. Studies	Comment
PubMed	(visual acuity) AND (randomised OR randomized) AND (wet age-related macular degeneration [Title/Abstract]) OR (exudative age-related macular degeneration [Title/Abstract]) OR (neovascular age-related macular degeneration [Title/Abstract]) OR (wet AMD [Title/Abstract]) OR (exudative AMD [Title/Abstract]) OR (neovascular AMD [Title/Abstract]) OR (wet-AMD [Title/Abstract]) OR (exudative-AMD [Title/Abstract]) OR (neovascular-AMD [Title/Abstract]) OR (wAMD [Title/Abstract]) OR (eAMD [Title/Abstract]) OR (nAMD [Title/Abstract])) AND (aflibercept [Title/Abstract]) OR (ranibizumab [Title/Abstract]) OR (bevacizumab [Title/Abstract]) OR (abicipar [Title/Abstract]) OR (brolucizumab [Title/Abstract]) **Filters: Clinical Trial; Humans**	207	No filter was applied for the doses, times at which visual acuity was assessed or the nature of the prospective design, and these aspects were treated manually
Cochrane Central Register for Controlled Trials ^a^	“age related macular degeneration” in Title Abstract Keyword AND “Aflibercept” OR “Ranibizumab” OR “Bevacizumab” OR “Brolucizumab” OR “Abicipar” in Title Abstract Keyword AND randomized in Title Abstract Keyword AND visual acuity in Title Abstract Keyword—in Trials (Word variations were searched)	229	The selection process was as carried out on studies found in Embase but not referenced in PubMed. No filter was applied for the doses, times at which visual acuity was assessed or prospective nature, and these aspects were treated manually
CliniclTrials.gov	“age related macular degeneration” in Title Abstract Keyword AND “Aflibercept” OR “Ranibizumab” OR “Bevacizumab” OR “Brolucizumab” OR “Abicipar” in Title Abstract Keyword AND randomized in Title Abstract Keyword AND visual acuity in Title Abstract Keyword—in Trials (Word variations were searched) **Filters: Terminated OR Completed**	68	Of these 68 trials, 60 were retrieved from PubMed or Embase. No filter was applied for the doses, times at which visual acuity was assessed or the nature of the prospective design, and these aspects were treated manually

^a^ Only studies referenced in Embase were selected.

**Table 2:** Included reports.

Reference	Study Name	Phase	N	Treatment Arm (s) of Interest	Regimen Comparisons of Interest	Outcomes of Interest
Dugel PU et al. 2020b [23] (Dugel, Koh et al. 2020)	HAWK, HARRIER	III	1459	Aflibercept 2 mg q8w vs. brolucizumab 3 or 6 mg q12/8w	q8 vs. q12/q8	BCVA CRT IVI (for brolucizumab only)
Kertes PJ et al. 2020 [24] (Kertes, Galic et al. 2020)	CANTREAT	III	466	Ranibizumab	Monthly vs. T&E	BCVA CRT IVI
Ohji M et al. 2020 [14] (Ohji, Takahashi et al. 2020)	ALTAIR	IV	458	Aflibercept	T&E (2w or 4w intervals)	BCVA CRT IVI
Staurenghi G et al. 2020 [25] (Staurenghi, Garweg et al. 2020)	OCTAVE	III	305	Ranibizumab	PRN (VA guided vs. VA and/or OCT guided)	BCVA CRT IVI
Gillies MC et al. 2019 [26] (Gillies, Hunyor et al. 2019)	RVAL	IV	559	Ranibizumab 0.5 mg vs. aflibercept 2.0 mg	T&E	BCVA CRT IVI
Guymer RH et al. 2019 [27] (Guymer, Markey et al. 2019)	FLUID	IV	690	Ranibizumab 0.5 mg	T&E (intensive vs. relaxed retinal fluid treatment regimen)	BCVA CRT IVI
Kertes PJ et al. 2019 [28] (Kertes, Galic et al. 2019)	CANTREAT	IV	526	Ranibizumab	Monthly vs. T&E	BCVA IVI
Mitchell P et al. 2019 [29] (Mitchell, Souied et al. 2019)	ARIES	IV	271	Aflibercept	q8w vs. T&E	BCVA CRT IVI
Nunes RP et al. 2019 [30] (Nunes, Hirai et al. 2019)	–	III	30	Ranibizumab vs. bevacizumab	PRN	BCVA CRT IVI
Semeraro F et al. 2019 [31] (Semeraro, Gambicordi et al. 2019)	–	Pilot	20	Aflibercept	PRN	BCVA CRT IVI
Wykoff CC et al. 2018 [32] (Wykoff, Ou et al. 2018)	TREX-AMD	III	60	Ranibizumab	Monthly vs. T&E	BCVA CRT IVI
Russo A et al. 2018 [33] (Russo, Scaroni et al. 2018)	–	Pilot	29	Ranibizumab	PRN	BCVA CRT IVI
Silva R et al. 2018 [34] (Silva, Berta et al. 2018)	TREND	III	650	Ranibizumab	Monthly vs. T&E	BCVA CRT IVI
Dugel PU et al. 2017 [35] (Dugel, Jaffe et al. 2017)	OSPREY	II	99	Aflibercept 2 mg vs. brolucizuamb 6 mg	q8w or q8w/q12w	BCVA CRT IVI
Feltgen N et al. 2017 [36] (Feltgen, Bertelmann et al. 2017)	RABIMO	IV	40	Ranibizumab 0.5 mg	q8w vs. PRN	BCVA CRT IVI
Gallemore RP et al. 2017 [37] (Gallemore, Wallsh et al. 2017)	RADICAL	II	82	Ranibizumab 0.5 mg		BCVA CRT IVI
Li K et al. 2017 [38] (Li, Chen et al. 2017)	SIGHT	III	228	Aflibercept	q8w	BCVA IVI
Mori R et al. 2017 [39] (Mori, Tanaka et al. 2017)	–	IV	58	Aflibercept	q8w vs. PRN	BCVA CRT IVI
Weingessel B et al. 2016 [40] (Weingessel, Mihaltz et al. 2016)	–	NR	16	Ranibizumab	PRN	BCVA CRT IVI
Berg K et al. 2016 [41] (Berg, Hadzalic et al. 2016)	LUCAS	NR	339	Ranibizumab 0.5 mg vs. bevacizumab 1.25 mg	T&E	BCVA CRT IVI
Berg K et al. 2015 [42] (Berg, Pedersen et al. 2015)	LUCAS	NR	371	Ranibizumab 0.5 mg vs. bevacizumab 1.25 mg	T&E	BCVA CRT IVI
Eldem BM et al. 2015 [43] (Eldem, Muftuoglu et al. 2015)	SALUTE	IV	77	Ranibizumab 0.5 mg	PRN	BCVA CRT IVI
Semeraro F et al. [44] (Semeraro, Russo et al. 2015)	–	Pilot	25	Ranibizumab	PRN	BCVA CRT IVI
Wykoff CC et al. 2015 [45] (Wykoff, Croft et al. 2015)	TREX-AMD	IIIb	60	Ranibizumab	q4w vs. T&E	BCVA CRT IVI
Ho AC et al. 2014 [46] (Ho, Busbee et al. 2014)	HARBOR	III	1100	Ranibizumab	q4w vs. PRN	BCVA CRT IVI
Dugel PU et al. 2013 [47] (Dugel, Bebchuk et al. 2013)	CABERNET	III	155	Ranibizumab 0.5 mg	PRN	BCVA CRT IVI (1-year data only)
Kodjikian L et al. 2013 [48] (Kodjikian, Souied et al. 2013)	GEFAL	III	374	Ranibizumab vs. bevacizumab	PRN	BCVA CRT IVI
Krebs I et al. 2013a [49] (Krebs, Vecsei Marlovits et al. 2013)	–	NR	24	Ranibizumab	PRN	BCVA CRT IVI
Krebs I et al. 2013b [50] (Krebs, Schmetterer et al. 2013)	MANTA	III	317	Ranibizumab vs. bevacizumab	Q4w	BCVA CRT IVI
Ranchod TM et al. 2013 [51] (Ranchod, Ray et al. 2013)	LUCE-DEX	II	20	Ranibizumab	PRN	BCVA CRT IVI
Heier JS et al. 2012 [52](Heier, Brown et al. 2012)	VIEW 1, VIEW 2	III	1815	Aflibercept vs. ranibizumab	q4w vs. q8w	BCVA CRT
Kaiser PK et al. 2012 [53] (Kaiser, Boyer et al. 2012)	DENALI	IIIb	112	Ranibizumab	PRN	CRT IVI
Larsen M et al. 2012 [54] (Larsen, Schmidt-Erfurth et al. 2012)	MONT-BLANC	II	133	Ranibizumab	PRN	BCVA CRT IVI
Soderberg AC et al. 2012 [55] (Soderberg, Algvere et al. 2012)	–	III	44	Ranibizumab	PRN	BCVA CRT IVI
Williams PD et al. 2012 [56] (Williams, Callanan et al. 2012)	–	Pilot	27	Ranibizumab	PRN	BCVA CRT IVI
Holz FG et al. 2011 [57] (Holz, Amoaku et al. 2011)	SUSTAIN	III	513	Ranibizumab	PRN	BCVA CRT IVI
Martin DF et al. 2012 [58] (Comparison of Age-related Macular Degeneration Treatments Trials Research, Martin et al. 2012)	CATT	III	778	Ranibizumab vs. bevacizumab	q4w vs. PRN	BCVA CRT IVI
Schmidt-Erfurth U et al. 2011 [59] (Schmidt-Erfurth, Eldem et al. 2011)	EXCITE	IIIb	88	Ranibizumab	q12w	BCVA CRT IVI
Vallance JH et al. 2010 [60] (Vallance, Johnson et al. 2010)	–	Pilot	9	Ranibizumab	PRN	BCVA CRT IVI
Dugel PU et al. 2020c [23]	HAWK/ HARRIER	III	1459	Aflibercept vs. brolucizumab	q8w vs. q8w/q12w	BCVA CRT IVI
Heier JS et al. 2011 [61]	CLEAR-IT	II	31	Aflibercept	PRN	BCVA CRT IVI
Kunimoto D et al. 2020 [62]	CEDAR/ SEQUOIA	III	1648	Ranibizumab vs. abicipar	q4w vs. q8w vs. q12w	BCVA CRT IVI
Khurana RN et al. [63]	CEDAR/ SEQUOIA	III	1411	Ranibizumab vs. abicipar	q4w vs. q8w vs. q12w	BCVA CRT IVI
The CATT Research Group, 2011 [64]	CATT	NR	1185	Ranibizumab vs. bevacizumab	q4w vs. PRN	BCVA CRT IVI
Taipale C et al. 2020 [65]		NR	52	Aflibercept	T&E	BCVA CRT IVI
Mitchell P et al. 2019 [29]	ARIES	IV	135	Aflibercept	T&E	BCVA CRT IVI
Li K et al. 2016 [66](Li, Zhu et al. 2016)	DRAGON	IV	499	Ranibizumab	q4w vs. PRN	BCVA CRT IVI

Subgroup of treatment-naïve patients; Selected study arm only (i.e., ranibizumab 0.5 mg q4w after ranibizumab induction). Abbreviations: NR, not reported; PRN, as-needed regimen; q4w, every 4 weeks; q8w, every 8 weeks; q12w, every 12 weeks; T&E, treat-and-extend regimen; BCVA, best-corrected visual acuity; CRT, central retinal thickness; IVT, intravitreal injections.

**Table 3:** Maintenance of changes in BCVA, CRT and IVI numbers over time using monthly, pro re nata and treat-and-extend regimens.

Change in Best-Corrected Visual Acuity (BCVA)					
Regimen	Year	N	Mean (ETDRS Letters)	95% CI	*p* Value
**Monthly**	1	11	8.8	[7.8; 10]	0.3494
	2	4	7.9	[6.5; 9.6]	
**PRN**	1	14	6.3	[5.4; 7.5]	0.9634
	2	5	6.3	[4.3; 9.1]	
**T&E**	1	11	7.6	[6.6; 8.8]	0.0905
	2	9	6.4	[5.5; 7.4]	
**Change in central retinal thickness (CRT)**					
**Regimen**	**Year**	**N**	**Mean**	**95% CI**	***p*** **Value**
**Monthly**	1	10	146.2	[131.1; 163.1]	0.0036
	2	2	185.9	[165.1; 209.4]	
**PRN**	1	17	118.7	[103.5; 136.1]	0.0005
	2	3	158.3	[144.8; 173.0]	
**T&E**	1	9	137.0	[122.4; 153.4]	0.9843
	2	8	136.8	[122.1; 153.3]	
**Number of injections**					
**Regimen**	**Year**	**N**	**Mean**	**95% CI**	***p*** **Value**
**Monthly**	1	6	10.6	[10.0; 11.3]	<0.0001
	2	2	22.9	[22.0; 23.9]	
**PRN**	1	18	7.3	[6.8; 7.9]	<0.0001
	2	2	13.3	[11.9; 14.9]	
**T&E**	1	13	8.2	[7.6; 8.8]	<0.0001
	2	9	14.6	[12.5; 17.0]	

Abbreviations: BCVA, best-corrected visual acuity; CRT, central retinal thickness; ETDRS, Early Treatment Diabetic Retinopathy Study; IVI, intravitreal injections; N, number of treatment arms; PRN, pro re nata (as-needed regimen); T&E, treat-and-extend regimen.

## Data Availability

To this end, a systematic review of published randomized studies was conducted from the MEDLINE and EMBASE databases and the Cochrane library.
